# Comparative efficacy and safety of contact force-sensing catheter and second-generation cryoballoon ablation for atrial fibrillation: a meta-analysis

**DOI:** 10.1590/1414-431X20176409

**Published:** 2017-08-07

**Authors:** X. Zhou, W. Lv, W. Zhang, Y. Ye, Y. Li, Q. Zhou, J. Zhang, Q. Xing, Y. Lu, L. Zhang, H. Wang, W. Qin, B. Tang

**Affiliations:** 1 Pacing and Electrophysiological Department, the First Affiliated Hospital of Xinjiang Medical University, Urumqi, Xinjiang, China; 2 Xinjiang Medical University, Urumqi, Xinjiang, China

**Keywords:** Atrial fibrillation, Ablation, Contact force-sensing catheter, Second-generation cryoballoon, Meta-analysis

## Abstract

This meta-analysis compared the efficacy and safety of the contact force (CF)-sensing catheter and second-generation cryoballoon (CB) ablation for treating atrial fibrillation (AF). Six controlled clinical trials comparing ablation for AF using a CF-sensing catheter or second-generation CB were identified from PubMed, EMBASE, Cochrane Library, Wanfang Data, and China National Knowledge Infrastructure. The procedure duration was significantly lower in the CB group compared with that in the CF group [mean difference (MD)=29.4; 95%CI=17.84–40.96; P=0.01], whereas there was no difference between the groups for fluoroscopy duration (MD=0.59; 95%CI=–4.48–5.66; P=0.82). Moreover, there was no difference in the incidence of non-lethal complications (embolic event, tamponade, femoral/subclavian hematoma, arteriovenous fistula, pulmonary vein stenosis, phrenic nerve palsy, and esophageal injury) between the CB and the CF groups (8.38 *vs* 5.35%; RR=0.66; 95%CI=0.37–1.17; P=0.15). Transient phrenic nerve palsy occurred in 17 of 326 patients (5.2%) of the CB group *vs* none in the CF group (RR=0.12; 95%CI=0.03–0.43; P=0.001). A comparable proportion of patients in CF and CB groups suffered from AF recurrence during the 12-month follow-up after a single ablation procedure [risk ratio (RR)=1.03; 95%CI=0.78–1.35; P=0.84]. AF ablation using CF-sensing catheters and second-generation CB showed comparable fluoroscopy duration and efficacy (during a 12-month follow-up), with shorter procedure duration and different complications in the CB group.

## Introduction

Atrial fibrillation (AF) is the most common sustained atrial arrhythmia, affecting over 5 million patients, and is often associated with dramatic adverse effects on quality of life and decreased survival (1,2). Drug therapy provides limited relief from arrhythmia and carries the risk of multiple side effects. Catheter ablation has been shown to be effective in decreasing recurrent atrial fibrillation. However, methods aimed at improving safety and efficacy of the technique are required (3). The novel cryoballoon (CB) and contact force (CF)-sensing catheter are revolutionizing the field of atrial fibrillation ablation (4).

*Contact force-sensing catheters*. Radiofrequency (RF) catheter ablation using CF-sensing potentially results in effective ablation. The catheter-tissue CF is measured at the catheter tip with an optical fiber or magnetic sensors. CF ablation for the treatment of AF is more efficacious than antiarrhythmic drug therapy with a lower rate of complications (5,6). Recent studies suggest that CF-sensing catheter usage reduces procedural time, and X-ray exposure (5,7,8).

*Cryoballoon*. Cryoballoon ablation is a standard approach for pulmonary vein (PV) isolation in symptomatic AF (9). The advantages of CB ablation include: reduced operator learning curve, the need for single trans-septal puncture, and minimal operator dexterity (10). Compared with the first-generation CB, the new Arctic Front Advance™ CB (second-generation CB) induces more homogenous and effective cooling at the surface of the balloon, which improves the efficiency of the procedure compared with the first-generation CB (11).

The purpose of this meta-analysis was to evaluate the efficacy and safety of AF ablation using CF-sensing catheter and second-generation CB.

## Material and Methods

### Literature search

Electronic databases, such as PubMed, EMBASE, Wanfang Data, China National Knowledge Infrastructure (January 1, 1998–February 1, 2016), and the Cochrane Controlled Trials Register for reports of all randomized controlled trials (RCTs) or controlled clinical trials (CCTs), were searched using the following medical terms: “contact force-sensing catheter”, "second-generation cryoballoon", “ablation”, and “atrial fibrillation” to capture data on AF ablation using CF-sensing catheters and CB. Abstracts of all identified RCTs or CCTs were independently screened by two authors, X. Zhou and W. Lv, to assess the relevance of the research question.

### Study selection and quality assessment

Studies fulfilling the following criteria were included: 1) patients receiving ablation for AF using CF-sensing catheters or second-generation CB; 2) patients with AF and/or paroxysmal AF (PAF) and/or persistent AF (Per AF); 3) human studies conducted in adults who were 18 years old and older. Non-comparative trials, case reports, editorials, and reviews were excluded from this study. Studies that did not report adequate outcomes of interest were also excluded.

The above authors independently assessed the validity and quality of the studies. The studies were individually checked for the following characteristics using a component approach: adequate sequence generation, allocation concealment, attrition less than 15%, blind assessment, intent-to-treat analysis, complete follow-up, and adequate AF monitoring.

### Data abstraction

The studies were reviewed and the data were abstracted independently by the above authors, and disagreements were resolved by discussion. Abstracted data included the following: 1) type of study, study size, study design, use of CF catheter or CB, and follow-up; 2) age, gender, detailed information pertaining to AF, PAF and/or Per AF patient subgroups, if available; 3) AF recurrence within 12 months; 4) complications including embolic event, tamponade, esophageal injury and transient phrenic nerve palsy, and 5) parameters related to safety, such as procedure duration and fluoroscopy duration.

### Statistical analysis

Statistical analysis was performed using the Cochrane RevMan version 5 software (The Cochrane Collaboration, UK). The results are reported as weighted mean differences and relative risk (RR) for continuous and dichotomous outcomes, respectively, with 95% confidence intervals (CI). The outcomes were pooled using the random-effects model when the heterogeneity was moderate or high (I^2^>50%). However, the fixed-effects model was used when the heterogeneity was low (I^2^<50%).The present study assessed the heterogeneity between studies using the Cochran’s Q statistic and the I^2^ index. All statistical testing was two-tailed with a statistical significance set at P<0.05.

## Results

**Figure 1.**
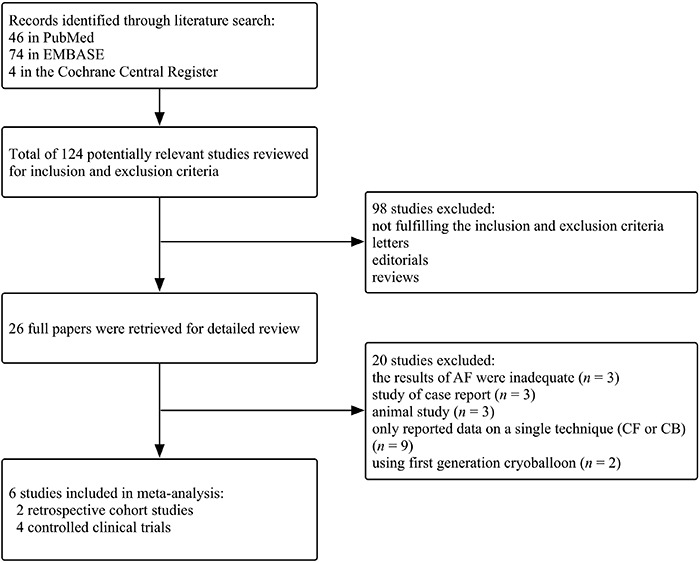
Flow diagram of the stages of the literature search. AF: atrial fibrillation; CF: contact force; CB: cryoballoon.

The electronic search identified 46 references from PubMed, 74 references from EMBASE, and 4 from the Cochrane Central Register of Controlled Trials. Among these 124 abstracts, 98 were excluded. The full manuscripts for the remaining 26 studies were retrieved for detailed review, and 20 were further excluded. Finally, 6 studies [12–17; 2 retrospective cohort studies (14,17) and 4 CCTs (12,13,15,16)] were identified for safety and efficacy of CF-sensing or second-generation CB in the setting of AF ablation. The data relevant to the literature search are shown in Figure 1.

### Publication bias

**Figure 2.**
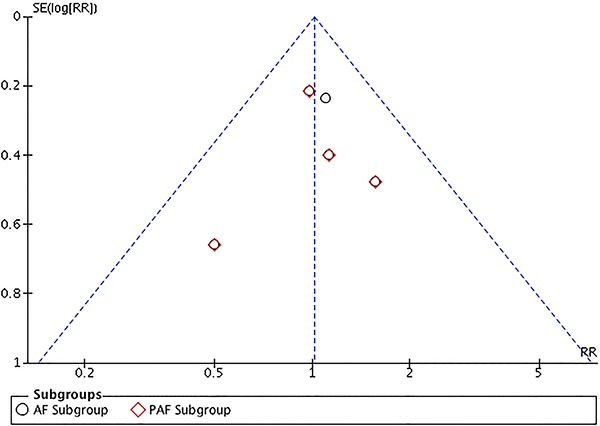
Funnel plot for assessment of publication bias in the primary outcome. Effect size is plotted on the x-axis and SE on the y-axis. AF: atrial fibrillation; PAF: paroxysmal atrial fibrillation; RR: relative risk; SE: standard error.

No significant publication bias was found for the primary outcome (AF recurrence at follow-up) as assessed by a funnel plot (Figure 2).

### Study characteristics

Supplementary Table S1 summarizes the characteristics of the included studies. A second-generation CB (Arctic Front Advance, Medtronic) was used in all the enrolled studies. Most of the studies had follow-up for a mean of 12 months, except Ciconte/Velagic et al. (14), which was a retrospective cohort study, and was included for procedure and fluoroscopy duration. Due to the nature of the study, it was not possible to blind the operator to the type of ablation protocol used.

It is noteworthy that two studies (13,14) focused on different patients. As shown in Supplementary Table S1, Ciconte/Velagic et al. (14) mainly focused on PAF patients, whereas Ciconte/Baltogiannis et al. (13) focused on Per AF patients. Although comprising different patient groups, the parameters were abstracted, without affecting the reliability of the current meta-analysis.

### Baseline patient characteristics

The baseline patient characteristics are also listed in Supplementary Table S1. A total of 739 patients were enrolled in both CF-sensing (n=387) and CB (n=352) groups. Five studies (12,14–17) provided detailed data of PAF patient subgroups, and relevant data was abstracted to compare the efficacy and safety in AF and PAF subgroups.

### Safety parameters

**Figure 3.**
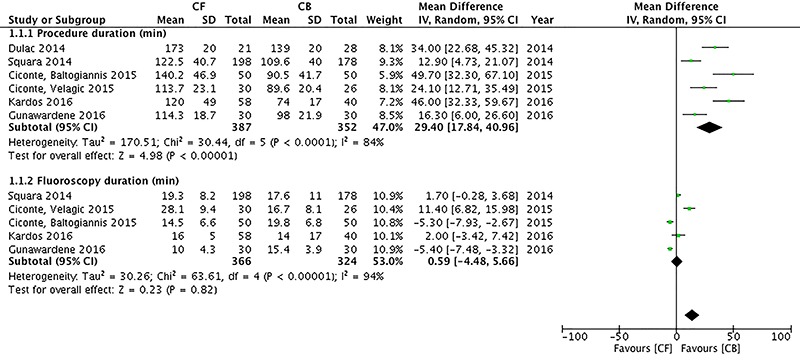
Forest plot showing unadjusted difference in the mean procedural duration of the contact force (CF) group compared with the cryoballoon (CB) group.

Figure 3 shows that the procedure duration was significantly lower in the CB group compared with the CF group [mean difference (MD)=29.4; 95%CI=17.84–40.96; P=0.01], which indicates that CB ablation could reduce procedural duration by a mean of 29.4 min compared to CF group. On the other hand, there was no difference in fluoroscopic duration between the CB and CF groups (MD=0.59; 95%CI=–4.48–5.66; P=0.82). Nevertheless, we found high quantified heterogeneity among studies for procedure duration and fluoroscopy duration, with the I^2^ values rising up to 84 and 94%, respectively.

### Complications

**Figure 4.**
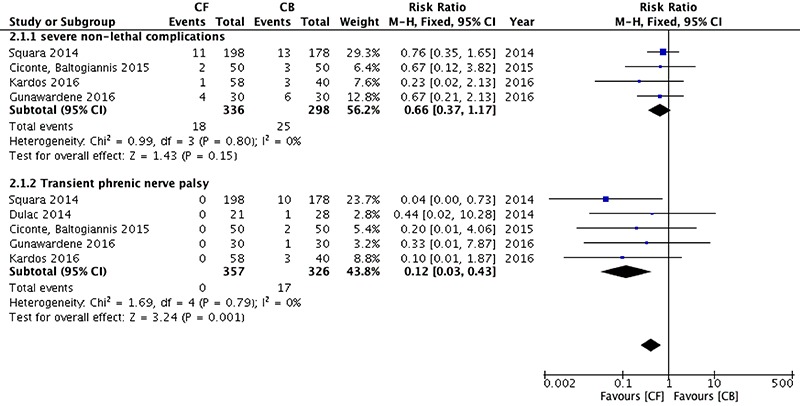
Forest plot showing the risk ratio and 95%CI in the incidence rate of complications among studies comparing contact force (CF) and cryoballoon (CB) groups.

As shown in Figure 4, different complications occurred in CF and CB groups, respectively. The complications were classified as in-hospital death, non-lethal complications and phrenic nerve palsy (which mainly occurred in cryoballoon ablation). Non-lethal complications included embolic event, tamponade, femoral/subclavian hematoma, arteriovenous fistula, PV stenosis, phrenic nerve palsy and esophageal injury. No patient died in either group. There was no difference in the incidence rate of non-lethal complications between CB and CF groups (8.38 *vs* 5.35%; RR=0.66; 95%CI=0.37–1.17; P=0.15). On the other hand, transient phrenic nerve palsy occurred in 17 of 326 patients (5.2%) of the CB group *vs* none in the CF group (RR=0.12; 95%CI=0.03–0.43; P=0.001).

### Long-term efficacy

**Figure 5.**
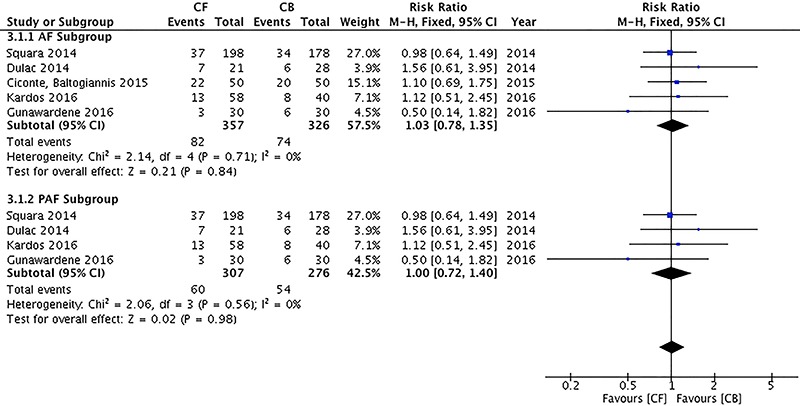
Forest plot showing risk ratios and 95%CI for atrial fibrillation recurrence within 12 months for studies comparing contact force (CF) and cryoballoon (CB) groups.

As shown in Figure 5, AF recurrence within 12 months was compared in the AF (5 studies, 12,13,15–17) and PAF (4 studies, 12,15–17) subgroups. In the AF subgroup, 82 (22.9%) patients in the CF category suffered from AF recurrence *vs* 74 (22.7%) patients in the CB category [risk ratio (RR)=1.03; 95%CI=0.78–1.35; P=0.84]. In PAF subgroup, 60 (19.5%) patients in the CF *vs* 54 (19.6%) patients in the CB category suffered from AF recurrence (RR=1.00; 95%CI=0.72–1.40; P=0.98). A comparable proportion of patients in both groups suffered from AF recurrence during the 12-month follow-up after a single ablation procedure.

## Discussion

### CB *vs* CF ablation

The meta-analysis demonstrated that second-generation CB ablation was not inferior to CF-guided RF ablation. The comparable success rate and procedural parameters suggest similar outcomes with both techniques in treating AF, irrespective of the energy source or the device. Therefore, the choice of energy source for AF ablation warrants attention (4). In most cases, the choice of RF or cryothermal ablation for AF often depends on the operator's skill, and on available material and associated costs (18). Jourda et al. (11) demonstrated that CB ablation in PAF is relatively safe and effective. Although phrenic nerve palsy has been described as the most frequent complication, the vast majority of patients recover quickly. The Fire and Ice trial (19) was a randomized evaluation of catheter ablation in patients with PAF. In this trial, CB ablation was not inferior to RF ablation with respect to efficacy for the treatment of patients with drug-refractory paroxysmal atrial fibrillation, and there was no significant difference between the two methods for overall safety. Nevertheless, the study was not powered to test the superiority of either the first-generation or the second-generation catheters. Our meta-analysis suggests that PAF patients who underwent ablation using either the novel Arctic Front Advance™ CB or the SmartTouch™ CF catheter present a similar likelihood of a recurrence-free condition at 12 months. More importantly, CB ablation is usually shorter and more reproducible than RF ablation in the setting of PAF (20,21).

Studies have recommended RF ablation for persistent AF, since additional linear or complex fractionated atrial electrogram ablation was not possible using the CB (15,18,22). The STAR-AF II trial (23) also suggested that PV isolation with RF alone might be a sufficient therapy for persistent AF.

### Incidence of late PV reconnection

Ciconte/Velagic et al. (14) reported higher PV reconnection rate after CF catheter ablation than second-generation CB ablation (1.8 *vs* 1.2 PVs/patient). The lower incidence of late reconnections following CB ablation might be due to larger and more uniform freezing zone on the balloon surface, leading to more homogeneous lesions than the traditional RF. The late PV reconnection was more frequent in veins with warmer nadir temperature and delayed time to isolation. A minimal temperature (<–51°C) predicted successful isolation without acute PV conduction.

### Procedure parameters

The current systematic review and meta-analysis demonstrated that the CB group had a tendency for a shorter procedure duration, whereas fluoroscopy duration was similar in both groups. In recent studies, procedural and fluoroscopy duration of CB procedures has been lower than in earlier studies. These findings might be explained by the cumulative experience with CB ablation at individual centers (13,20). Longer procedural and fluoroscopy duration in the CF group is probably due to the time required to create the 3D electroanatomical mapping and the point-by-point isolation of the PVs with RF ablation catheters (15). On the other hand, the shorter duration in the CB group might be attributed to single-step circumferential ablation procedure. Shorter procedural and fluoroscopy duration helps reduce the risk, as prolonged duration has been recognized as a risk factor for the development of effusions in AF catheter ablations (24).

The high heterogeneity among trials for procedure and fluoroscopy duration might be explained by the small sample sizes and the considerably discrepant cumulative experiences at individual centers.

### Mechanism of phrenic nerve injury caused by CB ablation

Phrenic nerve injury appears to be the most common complication during CB ablation with an overall incidence of 6.4% (4). The phrenic nerve injury caused by CB ablation may be due to the anatomic proximity of the right superior pulmonary vein to the phrenic nerve and cold transfer to deeper tissues during CB ablation. In addition, an undersized or a larger balloon also results in increased risk of phrenic nerve injury due to impingement of the phrenic nerve (9,25).

### Study limitations

The current analysis had the following limitations: 1) A few studies were of limited quality, given their retrospective and single-center designs. 2) Differences in operator experience and ablation protocols may have affected the outcomes in enrolled studies. 3) Some of the outcomes had a high I^2^ representing significant heterogeneity such as procedure and fluoroscopy duration, which may have limited the reliability of the current study although the random-effects model was used.

Our meta-analysis showed that both CF-sensing catheter and second-generation CB lead to comparable outcomes. There was no difference in the fluoroscopy duration between the CB group and the CF group, whereas the procedure duration was significantly lower in the CB group. Different complications occurred in CF and CB groups. There was no difference in the incidence rate of non-lethal complications between CB group and CF group. Further randomized and multi-centric evaluations are needed to confirm these preliminary results in order to identify specific subgroups more likely to benefit from one or the other technique.

## Supplementary Material

Click here to view [pdf].
